# Experimental glomerulonephritis induced by hydrocarbon exposure: A systematic review

**DOI:** 10.1186/1471-2369-6-15

**Published:** 2005-12-14

**Authors:** Uffe Ravnskov

**Affiliations:** 1 Magle Stora Kyrkogata 9, S-22350 Lund, Sweden

## Abstract

**Background:**

Much epidemiological evidence suggests that hydrocarbon exposure may induce glomerulonephritis and worsen its course in many patients. The mechanisms are unknown, however, no specific microscopic pattern has been identified, and it has also been argued that hydrocarbon exposure causes tubular damage mainly. Studying experimental animals may best answer these questions, and as no systematic review of glomerulonephritis produced experimentally by hydrocarbon exposure has been performed previously, I found it relevant to search for and analyse such studies.

**Methods:**

Animal experiments having mimicked human glomerulonephritis by hydrocarbon exposure were sought on Medline and Toxnet

**Results:**

Twenty-six experiments using thirteen different hydrocarbons were identified. Several human subtypes were observed including IgA nephritis, mesangial, proliferative and extracapillary glomerulonephritis, focal and focal-segmental sclerosis, minimal change nephropathy, anti-GBM and anti-TBM nephritis, and glomerulonephritis associated with peiarteritis nodosa. Glomerular proteinuria was seen in 10/12 experiments that included urine analyses, and renal failure in 5/8 experiments that included measurements of glomerular function. All experiments resulted in various degrees of tubular damage as well. In most studies, where the animals were examined at different times during or after the exposure, the renal microscopic and functional changes were seen immediately, whereas deposits of complement and immunoglobulins appeared late in the course, if at all.

**Conclusion:**

These experiments are in accord with epidemiological evidence that hydrocarbon exposure may cause glomerulonephritis and worsen renal function. Probable mechanisms include an induction of autologous antibodies and a disturbance of normal immunological functions. Also, tubular damage may increase postglomerular resistance, resulting in a glomerular deposition of macromolecules. In most models a causal role of glomerular immune complex formation was unlikely, but may rather have been a secondary phenomenon. As most glomerulonephritis subgroups were seen and as some of the hydrocarbons produced more than one subgroup, the microscopic findings in a patient cannot be used as a clue to the causation of his disease. By the same reason, the lack of a specific histological pattern in patients with glomerulonephritis assumed to have been caused by hydrocarbon exposure is not contradictive.

## Background

There is much observational evidence that exposure to organic solvents, paints, glues, fuels, motor exhausts and other environmental hydrocarbon contaminants may induce glomerulonephritis and also worsen renal function in a large number of patients [1-3]. Indeed, this hypothesis satisfies all except one of Hill's criteria for causality [3]. In spite of that the significance of hydrocarbon exposure has not been generally acknowledged and most current textbooks mention little if anything about this issue. Arguments often used by sceptics are that no probable mechanisms are known, that kidneys from animals exposed to hydrocarbons have shown tubular damage mainly, and that no specific glomerular pattern of hydrocarbon-associated glomerulonephritis has been identified in human beings. Glomerulonephritis has indeed been produced in a few experiments by exposing animals to hydrocarbons [3]. They are little known, and as no review of this subject has been published previously I found it relevant to perform a systematic search for such studies and found twenty-six.

## Methods

Using Medline and Toxnet I sought experiments that had produced glomerulonephritis by exposing animals to hydrocarbons. The search strategy included the formula (glomerulonephritis OR glomerulopathy) AND experiment* AND (hydrocarbon* OR solvent* OR X) where X was substituted by a large number of various hydrocarbons with putative toxicity and commonly used in the industry or elsewhere. Relevant papers were also sought in the reference lists of the studies. Papers that mentioned glomerular changes of any kind in the abstract were required as were papers without an abstract. All papers in the Western European languages were considered and included if appropriate.

## Results

Twenty-six experiments were identified, where the authors had noted microscopical changes in the kidneys of the animals similar to those seen in human glomerulonephritis after having exposed them to various hydrocarbons [4-28]. One experiment was reported in two papers [4,5], two groups used two different hydrocarbon [7,23]; totally 13 different hydrocarbons were used in 26 experiments. In two experiments [9,15] the animals were exposed to a single dose of the hydrocarbon, in the rest they were exposed intermittently. In 15 experiments, mainly the more recent ones [[6,9,11-14,16-18,22,24-27], 35], unexposed control animals or animals exposed to neutral substances were included. In all of them the renal changes, if any, were mild and did not exceed those seen in normal, aging rats. In ten experiments, the kidneys were examined by light microscopy (LM) only, in the rest by immunofluorescence microscopy (IM), and/or scanning or transmission electron microscopy (EM) also. Glomerular proteinuria was found in 10/12 experiments that included an examination of the urine. Evidence of renal insufficiency was found in 5/8 experiments that included a determination of renal function. In 19 studies the tubulointerstitial tissue were described also and in all of them varying degrees of damage were noted, in particular the two experiments that produced anti-TBM nephritis[19,20]. Findings similar to most of the human subgroups were seen, including IgA nephritis [18], mesangial [13,16], crescentic [6,14], proliferative [6,20] and focal segmental proliferative [19,20] glomerulonephritis, focal [18], focal segmental [17,22,27] and total [11,26] glomerular sclerosis, minimal change nephropathy [11,20,25], anti-TBM nephritis [19] and glomerulonephritis associated with periarteritis nodosa [9]. A summary of the findings is given in Additional file 1.

## Discussion

As shown in this review glomerular changes similar to human glomerulonephritis have been found in 26 expeiments by exposing animals to hydrocarbons, a strong support to the view, that such exposure may induce glomerulonephritis in human beings. The histological documentation in the early studies was cursory, but the more recent experiments, where the authors had examined the renal tissue by EM and/or IM also, have mimicked most of the human subgroups successfully. Few models of experimental glomerulonephritis using manipulation of the immune system have resulted in more harm to the kidneys than trace or transient proteinuria, unless they have included the use of Freund's adjuvant [29], the main ingredient of which is a mixture of hydrocarbon oils. In contrast, most of the experiments reviewed here, that included laboratory measurements, resulted in severe proteinuria and renal failure.

Up to five different subgroups of glomerulonephritis were seen after exposure to the same hydrocarbon. In some of the models it was obvious that they represented various stages of the disease. Genetic differences may also play a role, as many different animals were used. The most likely explanation is probably that the hydrocarbons used may not have been pure. Volatile hydrocarbons are purified by distillation, but as raw oil is composed of thousands of different hydrocarbons, many of which have a similar boiling point it is difficult to obtain a pure hydrocarbon. Therefore it is not possible to know whether it is the hydrocarbon, the name of which is printed on the bottle, that has resulted in a certain subgroup, or whether it has been caused by one of the impurities.

One of the arguments against the idea that hydrocarbon exposure may cause glomerulonephritis has been that a specific glomerular pattern has never been identified, but as almost all subgroups were found in these experiments this argument is obviously untenable.

The experiments offer several explanations regarding the mechanisms behind hydrocarbon-induced glomerulonephritis. Each of them may be sufficient by itself, although a complex co-operation seems more likely.

By combining with renal proteins hydrocarbons may act as haptens and induce autoimmunity against kidney cells. This mechanism was proposed by Nakajima *et al *in experimental DNCB nephritis [20] and may also have been operating in the experiment by Hartmann *et al. *[9].

A causal role of glomerular immune complex formation was unlikely in most of the studies because glomerular deposits of immunoglobulin and complement appeared late in the course, if at all and may therefore have been secondary phenomena without pathogenetic importance. This is in agreement with many clinical observations, where deposits of Ig and C in the glomeruli have been seen without evidence of renal disease [29]. Also, no study has ever found an association between degree of immune complex deposition or degree of glomerular damage, and the clinical course or outcome [29,30].

Hydrocarbons may influence T-cell functions leading for instance to an extrarenal production of cytokines with harmful effects on the glomerular epithelial and/or endothelial cells as in human minimal change nephropathy. This mechanism was likely in the experiment with maleic vinyl ether anhydride (MVE-2), diacetylbenzidine and carbon tetrachloride, as both podocyte fusion and proteinuria was seen immediately after the exposure [11,15,25-27] whereas immune deposits, if any, were seen much later [26]. In favour of an effect directed against extrarenal T-cells were the findings that pre-treatment with irradiation [25,26] or methylprednisolone [25] abrogated proteinuria, and also that radiolabeled MVE-2 was found to be located mainly to the reticuloendothelial system, not to the kidneys themselves [31].

The effects of hydrocarbons on the immune system are probably more complicated. Many hydrocarbons are able to suppress a wide range of immunological functions including the lymphoproliferative response to T-cell mitogens, memory cell and macrophage function, delayed hypersensitivity, serum immunoglobulin synthesis and susceptibility to infectious challenges, both in experimental animals and in man [32].

All experiments resulted in various degrees of tubular damage. Such damage may lead to an obstruction of the tubular flow, an increased postglomerular resistance and eventually a decreased glomerular filtration rate [33]. Together with an increased glomerular permeability these effects may result in a secondary trapping of circulating macromolecules. That proximal tubular damage may play a role in the causation of glomerulonephritis is suggestive also from the fact that most nephritogenic chemicals, for instance mercury, gold, silicium and lithium, are tubulotoxic as well, and that a large number of studies have found that the renal function and the course in glomerulonephritis is strongly predicted by the degree of tubulointerstitial damage, but not by the degree of glomerular damage [29].

The finding in one of the experiments that buffalo rats were susceptible to exposure, but not mice [7], and in another that female rats were more susceptible than male rats [6] confirms the experience from clinical studies that a genetic or sexual predisposition is necessary. This finding may also explain why many similar experiments have failed to produce glomerulonephritis.

The animal experiments are in accordance with the results from many cross-sectional and case-control studies. Thus, in 14 studies of workers exposed regularly to hydrocarbons significantly more had urinary findings typical of early glomerulonephritis than had age and sex-matched control individuals. The findings included a pathological urinary sediment, increased excretion of albumin and/or transferrin and/or glomerular antigens, and in three of them renal failure was also more prevalent [3]. And in a meta-analysis of 18 case-control studies odds ratio for exposure was 1.1 or lower in four studies that included early glomerulonephritis only, whereas in all studies that included patients with failure the ratio was 1.7 or, more often, much higher, in particular in studies that included patients with end-stage renal failure only [3].

## Conclusion

The successful imitation of human glomerulonephritis achieved in 26 experiments by exposing animals to various hydrocarbons is a further confirmation, suggested by numerous observational studies, that such exposure may induce glomerulonephritis in predisposed individuals. To convert this knowledge to benefit for the patients demands more research, however. Most important would be prospective studies of the effect of a discontinuation of the exposure. If the course in patients with renal failure could be reversed in this way, as was the case in a few retrospective studies and in a small prospective one [3], it would be the final proof that hydrocarbon exposure is causal in glomerulonephritis. At the same time it may become a major improvement in the prevention of terminal renal failure because hydrocarbon exposure is prevalent in patients with renal failure; in the four case-control studies that included patients with end-stage renal failure only, more than 50 % reported about significant exposure. As the microscopic findings obviously cannot be used as a marker for hydrocarbon exposure, such studies demand a thorough questioning of all patients with glomerulonephritis for possible environmental hazards, preferably in co-operation with experts in occupational medicine.

## Competing interests

The author(s) declare that they have no competing interests.

## Pre-publication history

The pre-publication history for this paper can be accessed here:



## Supplementary Material

Additional File 1 **Table 1**. Summary of methods, microscopic changes of the kidneys, and laboratory parameters of renal function in 23 experiments where animals were exposed to hydrocarbons. The author of this review has classified the glomerulonephritides. The tubular and interstitial changes are mentioned cursorily only. Click here for file
