# Supplementary material for: Experimental glomerulonephritis induced by hydrocarbon exposure: A systematic review
Source: BMC Nephrol. 2005 Dec 14;6:15. doi: 10.1186/1471-2369-6-15 (PMC1334181; doi:10.1186/1471-2369-6-15)
Supplement: Additional File 1 — Table 1. Summary of methods, microscopic changes of the kidneys, and laboratory parameters of renal function in 23 experiments where animals were exposed to hydrocarbons. The author of this review has classified the glomerulonephritides. The tubular and interstitial changes are mentioned cursorily only. [file 1471-2369-6-15-S1.doc]

**Table 1**. Summary of methods, microscopic changes of the kidneys, and laboratory parameters of renal function in 23 experiments where animals were exposed to hydrocarbons. The author of this review has classified the glomerulonephritides. The tubular and interstitial changes are mentioned cursorily only.

Abbreviations. LM: Light microscopy; IM: Immunofluorescence microscopy; EM: electron microscopy; h: hours; d: days; w: weeks; m: months; sc: subcutaneous; im: intramuscular; ip: intraperitoneal; bw: body weight; CIC: circulating immune complexes; GFR: Glomerular filtration rate; RBC: red blood cells; GBM: glomerular basement membrane; TBM: tubular basement membrane; C_in_: inulin clearance; PAH: para-amino-hippuric acid; PMN: polymorphonuclear leucocytes; C: complement; BUN: blood urea nitrogen

| **Author** | **A. Animal;**  **B. Dose;**  **C. Route;**  **D. Exposure time** | **Summary of microscopic findings and type of glomerulonephritis** | **Clinical chemistry** |
| --- | --- | --- | --- |
| **Xylene** | | | |
| Fabre *et al.* 1960 [10] | A. Wistar rats, rabbits  B. ?  C. Inhalation  D. 40-130 d | **LM:** Swelling of capillary tuft with cell desquamation and “inflammatory signs”. Necrosis of proximal tubular cells; RBC casts  **Not classifiable.** | Elevated urea, hematuria and albuminuria |
| **Bromoform; Dibromochloromethane** | | | |
| Condie *et al.* 1983 [23] | A. Male CD-1 mice  B. 0-289 mg/kg bw.  C: Oral  D: 2 w | **LM:** Enlargement and degenerative changes of the mesangium. Tubular damage. Changes of both glomeruli and tubuli were dose-related.  **Not classifiable.** | Renal cortical slice uptake of PAH decreased, but normal GFR |
| **Trichloroethylene** | | | |
| Nun 1938 [4]  Lande *et al.* 1939 [5] | A. Guinea pigs; rabbits  B. ?  C. inhalation, oral  D. 1-5 m | **LM**: Guinea pigs: Varying degrees of glomerular fibrosis and tubular degeneration. Rabbits: After 1 m tubular damage only. After 4 m “Glomérulite subaiguë” and tubular degeneration.  **Not classifiable.** | No data |
| Mosinger & Fiorentini 1958 [8] | A. Cats; guinea pigs  B. 1600 ppm  C: Inhalation  D. 10 h-10 m | **LM**: Swelling of Bowman´s space; Proliferation and enlargement of capillary tuft and parietal cells. Tubular damage; rare interstitial changes.  **Not classifiable.** | No data |
| Mensing *et al.* 2002 [34] | A. Male Long-Evans rats  B. 500 ppm  C. Inhalation  D. 30 h/w ; 6 m. | **LM**: “Perivascular, interstitial infections and glomerulonephritis could well be detected.”  **Not classifiable.** | LMW proteinuria, urine albumin normal. |
| Diacetyl-benzidine | | | |
| Harman *et al.* 1952 [6] | A. Sprague-Dawley rats B. 0.043 % of the food C. Oral  D. 3-12 w | **LM:** Proliferation of almost all glomeruli; epithelial hyperplasia; crescent formation. Tubular damage. Worse in female rats.  **Proliferative glomerulonephritis.** | Nephrotic syndrome |
| Dunn *et al.* 1956 [7] | A. Buffalo rats  B. 0.26 % of the food  C. Oral  D. 3-4 m | **LM :** Vacuoles within glomerular loops. Adhesions between tufts and Bowman´s capsule. Mild tubular damage. Many animals had no changes. Worse in female rats. No changes in mice. Similar findings after exposure to tetramethyl-benzidine.  **Not classifiable.** | Lipemia. |
| Bremner & Tange 1966 [12] | A. Wistar rats  B. 100-200 mg, single dose  C. sc, ip  D. 6-15 m | **LM:** 10-100% of glomeruli damaged. Dilatation of capillaries; thickening of capillary walls; reduced numbers of glomerular cells; finely granular eosinophil material in capillary walls; adhesions between tufts and Bowman´s capsule. Tubular damage.  **Not classifiable.** | Proteinuria. |
| Harman 1971 [14] | A. Sprague-Dawley rats  B. 0.043 % of the food  C. Oral  D. 1-36 w. Rats were examined weekly | **LM:** Focal proliferation and sclerosis with 80% crescents; vacuoles in parietal cells; mesangial proliferation; GBM thickening. Proximal tubular damage. Interstitial edema.  **Extracapillary glomerulonephritis; focal sclerosis** | Renal failure, nephrotic syndrome |
| Carroll *et al.* 1974 [15] | A. Sprague-Dawley rats  B. 200 mg, single dose  C. ip  D. 12, 16, 20 w | **LM:** Multiple cysts in glomeruli; occasional small crescents; glomerular tufts adherent to Bowman´s capsule. No proliferation.  **EM:** Cysts in visceral and parietal cells; fusion of foot processes; denudation and thickening of GBM. Occasional inflammatory cells in the capsular space. Tubules not described.  **Not classifiable. (Exsudative?)** | Nephrotic syndrome |
| Zimmerman 1979 [17] | A. Female Sprague-Dawley rats  B. 0.05-0.1 ml twice per w in 10 w  C. ip.  D. 1.5-35 w | **LM:** Segmental glomerular sclerosis; vacuolisation of visceral and parietal cells; increased mesangial matrix: synechia formation. Tubular damage; moderate interstitial nephritis.  **EM:** Vacuoles in epithelial and parietal cells; fusion of foot processes; no deposits. Thickened TBM.  **IM:** Occasional focal, segmental granular glomerular deposits of fibrinogen; no Ig or C.  **Focal, segmental sclerosis**. | Albuminuria; BUN normal |
| Dinitrochlorobenzene | | | |
| Floyd et al. 1975 [16] | A Male Wistar rats  B. 10 mg in benzyl-alcohol every 6. w  C. sc  D. 9 m | **LM**: Proliferation of mesangium and parietal epithelium; thickening of Bowman’s capsule; sparse infiltration of PMN; crescents in severe cases. Moderate tubular damage.  **IM**: Granular fibrinogen in glomeruli and Bowman´s capsule; minimal glomerular IgG; C3 sparse or absent.  **Proliferative (exudative?) glomerulonephritis**. | Heavy, unselective proteinuria. |
| Nakajima 1981 [19] | A. Hartley guinea pigs  B. 2μl 50% in acetone every 12. d, or 10μl once  C. sc  D. 3 w-2 m | **LM**: Focal, segmental proliferation. Tubular damage; interstitial nephritis.  **IM**: Linear IgG and C3 along TBM; sparsely and fragmented or granular along GBM, but not before d 12.  **Focal, segmental proliferative glomerulonephritis; anti-TBM nephritis**. | No data. |
| Nakajima et al. 1982 [20] | A. Hartley guinea pigs  B. 3μl 50% in acetone every 12. d  C. sc  D. 3 w to 2 m | **LM**: Minimal change or focal, segmental proliferation, mainly mesangial; thickening of GBM and Bowman´s capsule; periglomerular fibrosis. Interstitial nephritis; tubular damage.  **EM**: Fusion of foot processes; partial and irregular thickening of GBM with nodular deposition of material; opaque and linear deposits in TBM  **IM**: Linear IgG and C3 along TBM, sparsely and fragmented linear or granular along GBM. Same findings in kidney slices from non-exposed animals treated with sera from exposed animals.  **Focal, segmental proliferative glomerulonephritis; anti-GBM nephritis, anti-TBM nephritis**. | No data |
| White spirit | | | |
| Easley et al. 1982 [21] | A. C3Hf/Bd mice  B. 50μl.  C. sc  D. 3 times per w in 6 w | **LM**: “Chronic glomerulonephritis was seen in a few mice”  Multiple renal infarcts; papillary necrosis.  **Not classifiable**. | No data |
| Carbon tetrachloride | | | |
| Sakaguchi et al. 1964 [11] | A. Female Sprague-Dawley rats  B. 0.05 ml/100 g bw, twice per w  C. sc  D. 1, 2, 3 and 4 m | **LM**: Slight increase of mesangial matrix. Slowly worsening with thickening of GBM and progress to moderate glomerular sclerosis. Mild tubular damage.  **EM**: Initially fusion of foot processes and local GBM thickening. Eventually severe thickening of GBM with subendothelial deposits and increase of mesangial matrix. **Minimal change nephropathy → glomerulosclerosis** | No data |
| Gormly et al. 1981 [18] | A. Lewis rats  B. 10 min. 2-3/w  C. Inhalation  D. 5, 20 and 40 w | **LM**: Mildly, enlarged glomeruli. Liver cirrhosis in 10/15 animals  **EM**: Thickening of GBM, increased mesangial matrix and mesangial dense deposits. Tubules not described.  **IM**: Deposits, mainly mesangial, of IgA and C3, to a lesser extent IgG and IgM. The depositions increased with time  **IgA-nephritis → Focal sclerosis**. | Serum immunoglobulins and CIC increased, but only in rats with liver cirrhosis. No proteinuria. |
| Zimmerman et al. 1983 [22] | A. Female Sprague-Dawley rats  B. 0.05-0.1 ml daily  C. Oral  D. 25 and 36 w | **LM**: Focal, segmental and total sclerosis of glomeruli. Mild tubular changes, interstitial nephritis.  **EM**: Focal, glomerular hyalinosis and sclerosis; signs of necrosis in epithelial cells; fusion of foot processes. Electron-dense mesangial deposits. Later denudation of epithelial cells  **IM**: Granular segmental C3, IgM and fibrinogen; no IgA or IgG  **Focal, segmental sclerosis**. | Proteinuria.  Serum urea normal |
| Ogawa et al. 1992 [26] | A. Male Balb/c mice  B. 0.1 ml/kg bw biweekly  C. ip  D. 8 w. Examined 2, 7, 14, 42 and 84 d after the last exposure | **LM**: Initially no prominent glomerular changes. Tubular damage. Eventually, glomerular hyalinosis and marked interstitial nephritis. Transient liver damage..  **IM**: Mesangial IgG, but only at day 84, no deposits at d 14 and 42. No IgA or C3 at any time.  All renal changes were prevented by pre-treatment irradiation.  **Glomerulosclerosis**. | Proteinuria  Increased BUN |
| Ogata et al. 1995 [27] | A. Male Sprague-Dawley rats.  B. 0.5 ml/kg bw biweekly  C. Oral  D. 8w. Examined d1, w1, w2, w4 and w8 after the last exposure | **LM**: Early focal, segmental thickening of mesangium progressing to marked glomerulosclerosis; eosinophilic and PAS-pos droplets in podocytes and parietal cells; fusion of capillary loops.  **EM**: Early mesangial increase; partial fusion of podocytes; irregular thickening of GBM with subepithelial granules in a few glomeruli. Eventually severe alterations in almost all glomeruli.  **Mesangial glomerulonephritis; focal segmental sclerosis.** | No data |
| Trimethylpentane | | | |
| Norton & Mattie 1987 [24] | A Fischer rats  B. 1.5 ml/kg bw twice weekly  C. Oral  D. 1-4 w | **EM**: Marked increase in the number and size of epithelial microvilli. Proximal tubular damage.  **Not classifiable.** | No data |
| Petrol | | | |
| Klavis & Drommer 1970 [13] | A. Wistar rats  B. 20,000-40,000 ppm  C. Inhalation  D. 2.5 h per w in 6 w | **LM**: Focal proliferation of mesangial cells. Focal tubular damage; mild focal interstitial inflammation  **EM**: Partial fusion of foot processes, proliferation of mesangial cells, thickening of GBM.  **Focal, proliferative glomerulonephritis**. | No data |
| Maleic vinyl ether anhydride | | | |
| Bertolatus et al. 1988 [25] | A.Male Sprague-Dawley rats  B. 100 mg/kg bw  C. iv  D. Daily in 3 d | **LM**: Glomeruli and interstitium normal. Extensive cast formation correlated with degree of proteinuria.  **EM**: Fusion of foot processes; vacuoles in glomerular cells independent of degree of proteinuria, also in irradiated and steroid-treated rats.  **IM**: No IgG, IgM, C3 or fibrinogen.  **Minimal change nephropathy**. | Lowered C_in_, non-selective proteinuria. Abrogation of proteinuria by pretreatment with methylprednisolone or irradiation, but not with cyclosporine. |
| **4’-Fluoromethylbenzanthrene** | | | |
| Hartmann et al. 1959 [9] | A. Albino Holtzman rats  B. Single injection; 2.14 or 1.07 mg  C. sc.  D. Examined every week | **LM**. Hyaline necrosis of afferent arterioles and glomerular capillaries. Swollen glomeruli adherent to Bowman´s capsule. Regress after w 4-5  Periarteritis in all major organs, including the kidneys.  **Not classifiable**. **Periarteritis nodosa** | No data |
